# The Drivers of Environmentally Sustainable Hospital Foodservices

**DOI:** 10.3389/fnut.2021.740376

**Published:** 2021-10-15

**Authors:** Stefanie Carino, Shirin Malekpour, Judi Porter, Jorja Collins

**Affiliations:** ^1^Department of Nutrition, Dietetics and Food, Monash University, Notting Hill, VIC, Australia; ^2^Dietetics Department, Eastern Health, Box Hill, VIC, Australia; ^3^Monash Sustainable Development Institute, Monash University, Clayton, VIC, Australia; ^4^School of Exercise and Nutrition Sciences, Institute for Physical Activity and Nutrition (IPAN), Deakin University, Geelong, VIC, Australia

**Keywords:** foodservice, hospital, sustainability, food systems, planetary health

## Abstract

**Background:** Hospital foodservices have the potential to positively contribute to the local food system and planetary healthcare. Understanding the factors contributing to the success of “exemplar hospitals” with environmentally sustainable foodservices gives an opportunity to reimagine foodservices and guide strategic planning. The aim of this study was to identify the drivers of sustainable hospital foodservices.

**Methods:** For this qualitative multiple case study, purposive sampling was used to identify exemplar hospitals internationally. Semi-structured interviews were conducted with staff with extensive knowledge of their foodservices to explore the drivers of sustainable practices. Relevant documents provided background on the case. These documents and interview data were analyzed using the framework and thematic analysis.

**Findings:** There were 21 participants from 14 hospitals recruited across nine countries. Sustainable foodservice practices included local and organic food procurement, gardens onsite, vegetarian menus, re-serving unopened portion-controlled items, traditional foods, and food waste composting. Four themes were identified: initiating drivers, supporting enablers, challenges, and influence. Initiating drivers that “sparked” sustainable practices included the values of individuals or the hospital (e.g., community, environmental, or religious values), logical solutions to a problem, or government requirements. Enablers that facilitated success included motivated individuals, dedicated personnel, supportive leadership, internal protocols, and perceived benefits. External enablers included being part of member organizations, government requirements, and learning from other hospitals. Exemplar hospitals had broader influence, including educating the hospital community, supporting other hospitals, and influencing government policies/targets. Common challenges were staff resistance and inadequate policy directive.

**Interpretation:** These findings examine the successful international cases of sustainable hospital foodservices to provide a global overview to assist with strategic planning both within hospitals and within governing bodies.

## Introduction

Transitioning to planetary healthcare, described as environmentally sustainable health systems, is now more urgent than ever (1). Internationally, health services are increasingly taking action to deliver their services within planetary boundaries, with examples such as Kaiser Permanente achieving their goal of becoming carbon neutral in 2020 and the UK National Health Service's commitment to the net-zero greenhouse gas emissions by 2040 (2, 3). However, this positive change is not as widespread or consistent as it needs to be. As “anchor institutions,” hospitals can strategically utilize their position to have influence beyond the hospital, including promoting the health of staff and patients and supporting the local community and economy (4). Hospitals are an ideal setting for sustainability efforts, given the potential health professionals have as sustainability advocates and communicating the connection between environmental sustainability and health widely (5).

Reimagining foodservices in hospitals is a key part of the movement toward planetary healthcare. With consistently significant purchasing power, use of natural and human resources, and waste generation, foodservices play a vital role in the food system. Hospital foodservices can navigate a circular economy model to deliver better environmental and economic outcomes (6). In doing so, hospital foodservice can contribute to achieving the United Nations Sustainable Development Goals of “responsible consumption and production” (7). However, foodservice is often an overlooked aspect of healthcare delivery, food system, and dietetic research (8). To date, foodservice in hospitals has focused on delivering food to patients that meet their nutritional needs. There is now a need to transform the culture of hospital foodservices toward patient-centered care that prioritizes the health of the planet as well as promotes both healthy and sustainable diets for patients and the systems that deliver it.

In the research vision for food systems in the 2020s outlined by the editors of Global Food Security, it was put forth that behavior and systemic change are needed in food systems, and therefore, research should harness the perspectives of those working within the system and investigate innovation, policy, institutions, and practice (9–11). Previous research has identified discrete examples of hospitals that are implementing innovative environmentally sustainable foodservice practices; however, the drivers of their success are yet to be synthesized (12). Capturing and analyzing the stories of these hospitals would provide global information and lessons for broader transformations, both within hospitals and within their operational systems. Understanding the factors contributing to these hospitals having effective environmentally sustainable practices could provide the information needed to make recommendations for hospitals to improve their foodservices, policy, and best practice guidelines. Therefore, the aim of this study was to identify the drivers of environmentally sustainable foodservices and to develop a framework to operationalize environmentally sustainable hospital foodservices.

## Methods

This study was approved by the Monash University Human Research Ethics Committee (Project ID: 24912).

### Research Design

A qualitative multiple case study research design was used. As described by Stake, a multiple case study (also known as a collective case study) aims to understand a particular phenomenon through the study of multiple cases (13). The phenomenon under study was the driver of environmentally sustainable foodservice practices in hospitals. The multiple-case design allows for the examination of cases from multiple regions and a range of institutional environments, in order to derive overarching lessons and global insights into the issue under study (14). The DESCARTE (Design of Case Research in healthcare) model provides guidance in conducting high-quality multiple case studies in healthcare and so was used to guide study design and analysis decisions (15). The philosophical underpinning was pragmatism. In this way, the study was designed to provide the practical answers needed to work toward environmentally sustainable hospital foodservices (16). It draws upon the idea that reality is encountered, and knowledge is formed, through human experiences (17).

### Participants

Cases were selected using three criteria as outlined by Stake (13). First, the case had to be relevant to the phenomenon of interest (demonstrated environmentally sustainable hospital foodservices). Second, the cases must provide diversity, for example, a range of countries, types of hospitals, and sustainable practices. Third, they must provide an opportunity to learn about complexity and contexts, for example, a range of different factors that contributed to their success. Determining the number of cases was guided by the concept of information power (18). The sample size had sufficient information power when it fulfilled the study aims and included hospitals from a variety of contexts and with a range of different practices, as related to the study aim.

Purposive sampling was used to recruit international hospitals that demonstrated leadership and excellence in environmentally sustainable foodservice practices, at any stage of the food supply chain. They were identified through various mediums such as being featured in webinars about sustainable healthcare run by various sustainability networks, recommendations from colleagues, identified in literature or reports available online, or listed online as having received relevant awards, such as the Soil Association and Practice Greenhealth awards. Snowball sampling was also used whereby participants were asked if they knew of other hospitals that met the inclusion criteria. An advertisement for the research was also communicated through various sustainability networks (e.g., Global Green and Healthy Hospitals, Healthcare Without Harm) in Australia, Asia, and Europe.

Hospital contacts were emailed and invited to participate by nominating someone who works in the hospital with extensive knowledge about their sustainable foodservice practices to complete an interview. Participants obtained organizational permission from a senior hospital staff member and consented verbally at the beginning of the interview. Recruitment was not limited to solely English-speaking participants although contact was made with an email in English and interviews were conducted in English.

### Data Collection

Prior to the interview, the interviewer identified documents and information available online detailing the hospital's sustainable foodservices as background information. This was to become familiar with the hospital's context and practices, to use as prompts during the interview. Data were collected *via* semi-structured individual or small group interviews for cases with more than one suitable participant. Participants with English as their non-primary language often had a translator, invited a colleague proficient in English or used a translation program throughout the interview. The interview protocol (Supplementary Figure 1) was established by the research team and tested for content and sequence with a local foodservice dietitian and refined. To guide exploration of the institutional drivers existing at the hospitals, the “three pillars of institutions,” described by Scott being “regulative,” “normative,” and “cultural-cognitive” structures were used to guide prompts in the interview protocol (19). All interviews were conducted *via* a video conferencing program Zoom, with video and audio-recorded, between October 2020 and January 2021. The first author was the interviewer and was a clinical dietitian and researcher with prior experience in environmentally sustainable foodservice research.

Demographic data were collected verbally for descriptive purposes. Participant information included position title, years in the position, employment type, and gender. Hospital information included size (number of beds), type of hospital foodservice model (e.g., cook chill, cook fresh), type of food production (e.g., centralized), public, or private. When participants referred to reports or documents during the interview, they were asked to contribute copies of these to provide additional context for the analysis.

Grounded in pragmatism, there was a need for thoughtfulness and reflection throughout the inquiry process (20). Field notes were taken before, during, and after the interview to aid reflexivity throughout the process. Prior to the interview, this included notes on initial ideas of the hospital-based on materials already seen. During the interview, notes were taken according to the research question, and the interpretation was checked with the participant throughout the interview. Following the interview, notes were taken on initial impressions, themes, and documents to follow up. Impressions of the interviews were shared with the research team and this guided further adaptation of the interview protocol and recruitment strategies. This included reflections on the number and diversity of cases as to whether the sample had sufficient information power to cease recruitment.

### Data Analysis

The aim of data analysis was to identify general patterns across the cases in accordance with the research question. First, the audio recordings were transcribed by a speech-to-text transcription application, Otter.ai., then checked for accuracy by one researcher, allowing familiarization with the data. An inductive approach was used initially to create a framework for data analysis. Each author read through a transcript, and the team then discussed key themes to develop a framework of four broad categories. One researcher then applied this framework to all interview and report data. Data organized within these four categories were coded by one researcher, using the process for thematic analysis as described by Braun and Clarke (21). Two researchers then reviewed a subsection of these codes for consensus with the first researcher. Codes were then collated into themes, and two researchers reviewed these themes. Frequencies of each factor (drivers, enablers, influences) reported by hospitals were recorded and used in results reporting.

## Results

### Hospital Cases

There were 14 hospitals recruited across nine different countries. Hospitals ranged in size from 12 to 2,200 beds and varied in the type of foodservice model and production. The majority were public hospitals. Participants reported on a range of sustainable practices, across the food supply chain as detailed in Table 1.

**Table 1:** Hospital characteristics (*n* = 14 hospitals).

**Case**	**Location**	**Type**	**Public/private**	**Size (beds)**	**Foodservice model**	**Production**	**Environmentally sustainable foodservice practices**
1	Australia	General	Public	50	Cook chill	Centralized	Food waste sent to a farm; unopened pre-packaged items sent to the food rescue organization
2	Singapore	General	Public	800	Cook fresh	Onsite	Rooftop garden; meatless Mondays; electronic menu ordering system Food waste processed by digester and used in garden onsite
3	Taiwan	General	Private, non-profit	1,000	Cook fresh	Onsite	Garden onsite; local food procurement; plant-based menu; removal of plastic straws; food waste used in the garden onsite; waste oil used to produce soap
4	Australia	General	Private	170–700	Cook fresh	Onsite	Room service model; food waste processed by dehydrator and sent to a waste-to-energy facility; allow local businesses to provide their food waste to be sent to dehydrator; leftover food donated to a local charity
5	Australia	General	Public	450	Cook fresh Cook chill	Combination—onsite and centralized	Oral nutritional supplements and portion controlled items unopened by patients are re-served; reusable packaging for bulk food purchased; plastic straw minimization; menu review focused on the minimization of high food waste items
6	Canada	Women and children's	Public	420	Cook fresh	Onsite	Local and organic food procurement; room service model; food waste composted; farmers market onsite
7	Canada	General	Public	80–450	Cook chill	Centralized	Local food procurement; vegetarian menu; room service model
8	Denmark	General	Public	600	Cook fresh	Onsite	Herb garden onsite; organic food procurement; menu based on EAT Lancet diet recommendations; food waste reduction strategies; donate unused food to the local community group
9	US	General	Non-profit	25–460	Cook fresh	Onsite	Local and sustainable procurement; farmers market onsite; food waste sent to farm; tokens for complimentary vegetables given to patients identified at risk of food insecurity; land donated for community gardens
10	Austria	General	Public	600–2,200	Cook fresh	Onsite	Organic food procurement; seasonal menus; meat reduction; food waste measurement
11	Taiwan	General	Private, non-profit	389	Cook fresh	Onsite	Local and seasonal food; vegetarian menu; food waste reduction strategies; food waste sent to a farm; cooking classes
12	UK	General + women's	Public	18–825	Cook fresh Cook freeze	Combination—onsite and centralized	Herb garden onsite; local/sustainable procurement; seasonal menu; no single-use plastics
13	Canada	General	Public	12	Cook fresh Cook chill	Combination—onsite and centralized	Traditional Indigenous foods served to patients
14	Netherlands	General	Public	635	Cook chill	Centralized	Menu redesign focused on food waste reduction and improved patient nutrition; sustainable, local purchasing; coffee grounds used to grow mushrooms

### Participants

Participants (*n* = 21) had been in their role for an average of 7.5 years, ranging from 0.5 to 20 years. The majority (90%) were employed full time and more than half (57%) were female. Foodservice staff was the most common, followed by dietitians and various departmental staff, as detailed in Table 2.

**Table 2:** Participant demographics (*n* = 21 participants).

**Demographic characteristics**	**Response**	***n* (%)**
Role	Foodservice/hospitality/catering staff Dietitian Hospital executive Sustainability staff Nutrition department director Health promotion director Community member with specialized knowledge Sustainable food coordinator	8.5 (40) 5 (24) 2 (10) 2 (10) 1 (5) 1 (5) 1 (5) 0.5 (2)
Years in role (years)	0–5 5–10 10–15 15–20	12 (57) 3 (14) 4 (19) 2 (10)
Employment status	Full time Part time	19 (90) 2 (10)
Gender	Female Male	12 (57) 9 (43)

**Figure 1:**
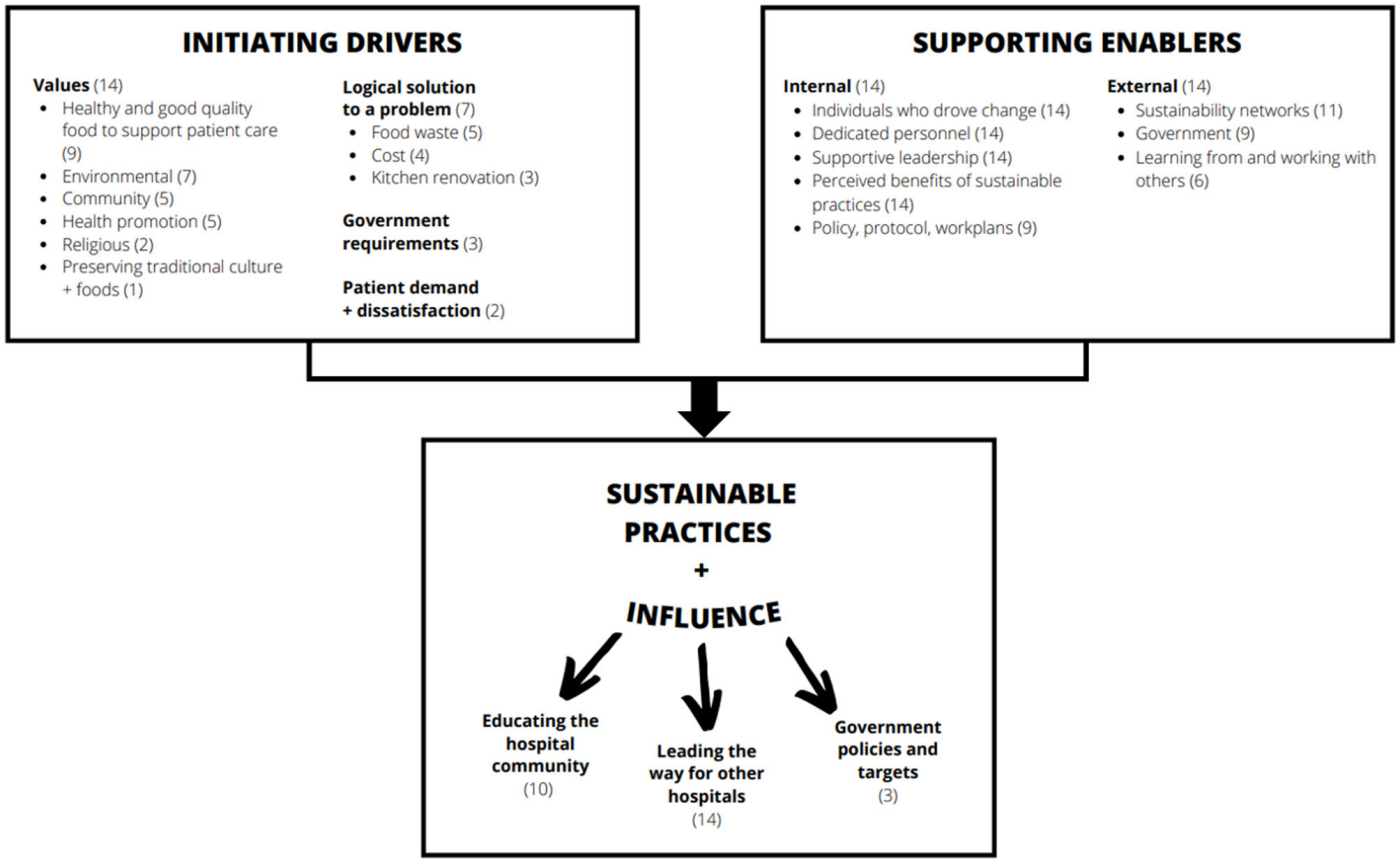
A framework of factors contributing to environmentally sustainable foodservice practices in exemplar hospitals and their wider influence (numbers reported represent the number of cases that reported the factor).

There were four themes identified in the results that described the functioning of environmentally sustainable foodservices. These were initiating drivers, supporting enablers, challenges, and influence. Figure 1 illustrates a framework of factors contributing to environmentally sustainable foodservice practices, as derived from the data.

### Initiating Drivers

This theme has considered triggers for hospitals to take action toward sustainable practices. These factors varied greatly among hospitals and depended on the cultural, social, and political contexts in which hospitals were operating. It was often patient dissatisfaction, values, and sustainable practices being seen as a logical solution to a problem, which worked together as initiating drivers of change.

#### Values

The values that existed were either established from the beginning of the hospital and embedded within its mission and vision or had been developed over time. These values were dependent on the hospital context, for example, hospitals that valued vegetarian diets due to religious beliefs, hospitals with strong ties to their local Indigenous communities, or those that valued high-quality, healthy, and sustainable food to support patient recovery.

“*We are convinced that organic food, because we are a children's hospital, kids need to have the best because they are growing, and they are really sick when they are here. So for us it was really a good objective, you know, to give them the best.” (Case 6)*

#### A Logical Solution to a Problem

Sustainable practices were often seen to be the logical solution to a preexisting problem. For example, high food waste often resulted in changing to a different foodservice model to increase food intake by patients. Renovations to the kitchen also presented as an opportunity to recreate the foodservice model.

#### Patient Demand and Dissatisfaction

Responding to patient dissatisfaction with the food provided was another underlying driver. This was related to aspects such as food quality or variety.

#### Government Requirements

There were few cases where changes were initially driven by government requirements for local or organic food. These hospitals, however, were driven to exceed these set requirements as a result of their values.

### Supporting Enablers

Supporting enablers were internal and external factors that helped a sustainable practice to come to fruition after it was triggered by an initiating driver.

#### Internal

##### Individuals Who Drove Change

In every case, there was an individual who was crucial to driving change. There were several characteristics that allowed these individuals to achieve success. Generally, these individuals had intrinsic motivation, which drove them to be self-directed, autonomous, and persistent despite the challenges they encountered. While there was only one example of an individual with a dedicated role in sustainable food systems, many were working toward practices that were outside of their traditional role, but they saw it as something that was important for them to achieve. This was often supported by senior staff.

“*I think that I've had leadership support to work outside of a really tight job description but really like when you look at doing healthy food work on this island. I mean it just, if you're not doing work with traditional food I don't think that you're doing your job” (Case 13)*

Individuals had relevant previous education or training not typical for their roles, such as public health or nutrition, or had a prior experience that meant they brought a different lens to the role. For example, foodservice managers that were previously chefs in restaurants were motivated to improve food quality. These individuals had existing relationships that were important for success. These relationships were within the hospital, with people in various departments, as well as external to the hospital such as peers in similar roles at other hospitals or sustainability networks. These individuals demonstrated leadership qualities in the way they worked to engage relevant staff in having a shared vision for sustainable food, for example, through using design thinking to build solutions together or providing a farm tour for kitchen staff to learn about local food. Managers ensured that their staff was comfortable to share their ideas and solutions and they would be listened to.

“*They really didn't seem to kind of grasp the concept initially until I took them off site and we had an opportunity. They met the farmers. So the distributor brought in the farmers that day that we spent there. The farmers could talk to them a little bit about their farms and the process and you know why it was important, you know, for them to partner with institutions like hospitals to provide produce and so forth so we had one farmer there he was a potato and carrot farmer. And we happen to be using his products all along and we didn't know. So the staff got really excited when, when they started to hear about you know what we were doing to support our local economy.” (Case 7)*

##### Dedicated Personnel

Several groups of people were involved in getting the necessary work done. There were working groups and committees dedicated to progressing sustainability ideas. Groups consisted of staff from various departments, and they demonstrated motivation. These groups allowed staff members to feel a sense of ownership, and there were mechanisms in place, such as work plans and performance indicators, to hold members accountable for following through with their tasks. There were many examples of effective collaboration between departments, in particular when a practice required several departments to change their tasks. A common collaboration was with health promotion or community health.

“*No pun intended, but it was kind of an organic growth out of, you know, just our culture. And it was kind of neat to see that it rose from different parts of the organization, you know, so at one hospital, it maybe came from our food service department, and another hospital that came from our, our health and wellbeing group” (Case 9)*

Through establishing new sustainable practices, shaped positive staff culture and contributed to a sense of pride, honor, and work satisfaction.

“*It has been a place where people come in the mornings with pride, to actually exercise their skills so it's a whole other way of being a professional” (Case 8)*

When specialist knowledge was needed, help was sought, for example, Indigenous cooks to prepare traditional foods authentically. Several hospitals referred to having community engagement that assisted their achievements. For example, community volunteers to help with gardening, or contribution of knowledge by local elders. In many cases, this ethos and values became embedded in the hospital's identity and staff culture.

Participants described that students or interns assisted with audits for local food, food waste, or disposable service ware. Volunteers assisted with gardening, and healthcare staff were drawn in to contribute.

##### Supportive Leadership

Leadership support was provided in two different ways. In some cases, senior staff initiated practices, or became involved with sustainability networks, or created dedicated roles. In other cases, where individuals had ideas, senior staff provided support and approval, however, did not play an active role in its achievement. There was the notion that drive and support were required from the top as well as the bottom to drive success.

“*I think it's important to have a senior leader being your advocate for the program. Without the senior leader advocate, you're not going to have success. And, you know, kind of the corollary with that, is that, you know, having support of the grassroots, the frontline staff, now those go hand in hand, you know, so having support at the top, and also, you know, the frontline staff that really are passionate about it.” (Case 9)*

Leadership buy-in was often the result of opportunistic timing attributed to several other factors. These factors included plans for the building of new facilities, positive staff culture, and demand for increasing sustainability work due to work being done in other departments of the hospital. Demand from the staff was also linked with an increase in media coverage on waste and climate change issues.

##### Perceived Benefits of Sustainable Practices

There were often multiple benefits to sustainable practices, for example, saving money associated with purchasing less meat or reducing organic waste going to landfills. Where funding was required, for example, to change to a different foodservice model, there was a general attitude that the investment would pay off. This was also the case for fees associated with joining sustainability networks, as it was seen to be a worthwhile investment considering the benefits that joining would provide. Many of the practices were seen as a logical solution to a problem and improved efficiency, for example, using reusable crates rather than staff having to break down cardboard boxes daily. In instances where food waste was composted rather than sent to landfills, this workflow process change was easy for staff to follow. Some practices made sense for the organization, being something that had previously been done. When positive feedback was received from patients or staff, this acted as a driver to continue and build upon the practice.

##### Policy, Protocols, Work Plans

Several cases created internal documentation to ensure that initiatives were embedded into an ongoing practice. These local protocols or decision pathways were multipurpose, serving to provide permission for local food purchasing or to instruct staff to follow a pathway for re-serving unopened oral nutrition supplements, for example. These documents also provided a sense of accountability for staff responsible for particular actions. Individuals acted as advocates to incorporate sustainability considerations into internal policies.

“*I thought right we need something written in writing that's going to be easily accessible to anybody who needs this so that in the future, if there's a change in food service dietitians or directors. We know that it's been approved.” (Case 5)*

#### External

##### Sustainability Networks

In the majority of cases, sustainability networks were major supports to the hospital's success. These included Nourish, Global Green and Healthy Hospitals, Health Promoting Hospitals, Practice Greenhealth, United against Waste, Healthcare without Harm, and Soil Association. They offered guidance on priority areas for action, ideas, networks of like-minded individuals, information/data sharing, resources, benchmarking, and auditing.

“*We met other people from Canada, I realized because I was the one who had that opportunity, you know, to meet others. I realized that other programs did great things, and it gave us the dream to do something else.” (Case 6)*

These organizations often had awards that incentivized hospitals to reach certain targets to receive recognition and promote their work.

##### Government

In less than half of the cases, there were government policies or incentives, which acted as drivers. For example, incentives to reduce organic waste to landfills, the city signing local government instigated food pledges or local food procurement policy, and law, which supports donation of leftover food.

In several instances, the government had provided grant opportunities, which provided the initial cash injection to establish sustainable practices.

##### Learning From and Working With Others

Many hospitals learned from others, including hospital and non-hospital settings, for example, through information sharing between chefs from different hospitals. Individuals working solely in a role aiming to create change sought support from those at other hospitals where there wasn't support internally. Collaboration with universities enabled research to be conducted, such as modeling a nutritionally adequate plant-based menu and waste audits, as well as recruiting students to assist with data collection.

Many hospitals worked with suppliers and group purchasing organizations to provide the ingredients they desired. They negotiated their contracts to embed these into ongoing purchasing, for example, a requirement for the central production kitchen to use local foods or communicating with group purchasing organizations a desire for increased availability of plant-based proteins and sustainably produced ingredients.

### Challenges

Exemplar cases reported several challenges experienced during the implementation of sustainable food practices.

#### Staff Resistance to Change

Resistance from the staff was reported for a range of reasons. Often changing to a different foodservice model meant that staff had to take on new or different tasks, which was not always received well. Although in many cases, leadership support was an enabler, there were instances whereby people in leadership or influential positions were perceived to be set in their ways and not open to making changes. There were some cases where it was difficult to gain leadership approval due to misconceptions around funding required to make changes, for example, in changing to locally procured foods.

“*When I first came to the hospital I bought a book and gave it to three of my managers. The book was called Who Moved My Cheese. And it's a small book, and it's about change. And I gave it to all our managers and I said read that. Because when you work in institutions like the* <*name of institution*>. *They are very, very slow to change. People have been working there for 20, 30, 40, 50 years in the same role. Change is difficult, but that Who Moved My Cheese is absolutely fantastic book because it really changes you changes your thoughts on change. But the two people. Two of them are still there, ones gone, they just didn't buy into that change at all. But I can't always make people listen and some people just don't want to listen.” (Case 12)*

There was often a lack of funding or time dedicated to knowledgeable staff on progressing sustainable food practices. This related to personnel with the foodservice skills required for a change in the production model, or knowledge on preparing traditional Indigenous food. There was difficulty gaining approval for the initial funding required to implement a change that would only have economic benefits in the long term.

#### Inadequate or Conflicting Policies and Standards

A lack of, or inadequate, internal policy was seen as a barrier to pushing forward sustainable practices. Purchasing restrictions and group purchasing organizations were often unsupportive of sustainable practices, for example, in limiting the hospital's ability to purchase local or traditional foods. Values of staff involved in setting buying lists and hospital staff purchasing goods differed and did not meet the quality or sustainability criteria desired by the hospital.

Where there were relevant purchasing policies, this conflicted with supplier contracts. For example, it was challenging to identify suppliers that met the sustainability criteria which hospitals were seeking. There were sustainability policies that were perceived to be too ambiguous or vague to be practical and were not necessarily monitored. A policy may have applied broadly for a city, but was not specific for the hospital setting with targets or criteria for guidance. Standards were not always mandatory or monitored, which was a challenge to ensure that staff prioritized their achievement.

There were standards for infection control and food safety, which limited sustainable practices. It was a lengthy process to identify a staff member to consider their ideas and work around these restrictive processes while still ensuring safety.

There were a few cases that reflected on the delay that the COVID-19 pandemic had put on their progress or had changed what they were able to do, for example, in foodservice projects.

### Broader Influences Beyond Environmental and Economic Outcomes

The influence of these exemplar hospitals was evident across three levels, within the hospitals' community, among other hospitals and policy/targets.

#### Educating the Hospital Community

Hospitals educated and engaged patients in sustainable food through menus with information about the food, for example, where it was produced, whether it was organic. There were also examples of hospitals providing recipes to use local produce, and having talks and awareness sessions. The goal of this was to trigger a change in personal lives of patients for when they go back home. This was common in hospitals that were part of the Health Promoting Hospitals network.

Hospitals strived to educate the wider hospital community around sustainable food. They did this by providing giveaways to staff such as dehydrated compost produced onsite, awareness days, staff challenges, resources such as recipe cards, and farmer's markets onsite with producers. Several hospitals reported working closely with their media and communications department to share their achievements. This helped to raise awareness in local communities of the work of the hospital and share sustainable food messages.

#### Leading the Way for Other Hospitals

Many hospitals saw themselves as trendsetters in sustainable foodservices, which inspired other hospitals. There were several mechanisms by which hospitals were able to share their experiences and inspire others.

Several of the individuals who drove changes were also active members of various networks and held leadership positions. Being part of sustainability networks allowed hospitals an opportunity to share their story and mentor other hospitals. It also provided an avenue to learn from other hospitals.

These hospitals also sought out and were invited to speak at conferences and as part of sustainability networks. This was also seen as an opportunity to influence personal views of their peers; for example, one hospital hosted a conference and ensured they provided tasty vegetarian meals to nudge the attitudes of attendees to consider introducing vegetarian menus at their hospital.

Several reported receiving awards through various organizations. The approach to these awards varied among hospitals, for some they inspired the hospitals to make changes in order to receive the awards, and the recognition was a motivation to keep achieving. For others, it was seen as valuable in raising the profile of the hospital and a platform for sharing their story.

Several hospitals created their own internal policy or statements that they shared with other hospitals. For example, an internal protocol outlining the steps to re-serve unopened oral nutrition supplements was shared with other hospitals interested in setting up similar protocols. The research was seen as an important way to promote healthy and sustainable diets. The capacity for research was both within the people involved and supported by research teams at the hospital.

#### Government Policies and Targets

The interrelationship between hospitals and government policy/targets was bidirectional. Some cases described their commitment to exceeding predefined government targets (e.g., for use of organics or waste management targets), while others shared their experiences in nudging the development of local and state-level policy and legislation. One facility successfully did this by inviting ministers to the hospital to promote their work, and wrote letters to members of parliament, and shared their own policy statements. This resulted in a sustainable food purchasing policy for all hospitals in the region. In other cases, they referred to their increasing reputation as indirectly influencing government and raising their awareness around food sustainability initiatives.

Several cases saw the value in influencing external organizations that were crucial to achieving sustainable food practices, such as suppliers and group purchasing organizations. They worked to engage them and work toward a solution together.

## Discussion

This research sought to identify the drivers of environmentally sustainable foodservices in hospitals around the world. The findings provide a global overview to support strategic planning and decision making for sustainable practices. The framework (Figure 1) illustrates there is an initiating driver that sparked action, and internal and external supporting enablers that allowed the change to be realized. The specific drivers and enablers varied across cases and existed in unique combinations dependent on context. Challenges relating to staff resistance and absent or inadequate policy were navigated. These hospitals had three key influences beyond reducing their own environmental and economic impacts, including educating the hospital community, role modeling for other hospitals, and influencing government policies and targets.

Considering Scott's Pillars of Institutions, the normative pillar, defined as the attitudes and values of the hospital and the people working within them, was prominent in driving sustainable practices (19). Much the same as previous literature, this study has found that sustainability efforts, when first formed and operating in a niche environment, are reliant on individual frontrunners rather than a widely agreed-upon consensus (22, 23). A combination of emergent and assigned leadership is crucial to success (24). An explanation for the success achieved by the individuals has been previously described as being based on their opportunity to influence the change, being prepared for the change, and valuing the change (25). Therefore, opportunities are needed to identify and nurture emerging leaders. Professional development needs to be provided and individuals need to be supported to seek out high-quality learning relating to sustainability, for example, those provided by the Center for Sustainable Healthcare in the UK and other sustainability networks (26). These topics must also be embedded within high-quality sustainable healthcare education to prepare the future workforce. Leadership and collaboration to incorporate sustainable healthcare into course accreditation standards will support this (27). A framework by Guzmán et al. has been described for planetary health education, which transcends competencies and encompasses the interconnection within nature, the Anthropocene and health, systems thinking, equity and justice, and movement building and systems change (28). While there were few examples of hospitals with a role dedicated to environmentally sustainable foodservices, the direct outcomes, both in the short and in the long term, of dedicated staffing have yet to have been documented. This is an area that hospitals that do have a dedicated role should endeavor to disseminate widely. Despite the value in empowering individuals to act, this solution is a symptom of the lack of the topic being widely discussed, valued, and acted upon in healthcare. In order to promote sustainable foodservices, and embed these principles within healthcare delivery more broadly to shape the wider food system, a system-level approach is needed (29). This approach must encompass higher education, professional development, standards of practice, clinical and community health, community engagement, organizational policy, and policy advocacy (29).

Healthcare systems are complex systems consisting of practices, roles, and organizational arrangements that can remain stagnant and restrict behavior and systems change over time (30). The exemplar hospitals described in this research were able to break this cycle and reimagine their foodservices. The mechanisms they used were similar to those previously identified in the literature on behavior and practice change interventions in complex healthcare settings. For example, audits and feedback, educational outreach visits, and patient-mediated interventions, particularly when used together, seem to be effective in forming the social systems to promote change in behavior norms (31). The enablers identified also align with literature on implementing best practices, in particular, leadership, champions, financial resources, staffing, and culture (32). However, relying on behavior change of individuals only goes so far, and this focus needs to shift to the systems that individuals operate within (33). These findings have practice implications for the way that we design and establish structures within hospitals that are conducive for the behavior change and effective implementation needed to achieve sustainable foodservices.

These exemplar hospitals have revealed an interesting dialogue around the role of policy. While previous research identified that those working within hospitals believe government policy is needed, this was not identified as a prominent driver in this study (34). Instead, in the absence of policy, sustainability networks provided guidance, as well as resources and networks to fill the gaps. Sustainability networks are an area previously identified as being beneficial in supporting healthcare professionals in addressing the challenges of climate change (35). Government policies and targets are needed that are specific and actionable that hospitals are able to realistically follow them. The question remains as to the power of policy in hospitals that are in the earlier phases of the “Waves of sustainability” model. This model describes the phases that organizations move through in supporting ecological sustainability (36). The early stages are active resistance, then move toward compliance, and finally a strong commitment to establishing sustainability values within the organization and wider society and industry, which was evident in the majority of cases included in this study. A policy might spark organizations to progress to the “compliance” phase. The internal and external structures in place could enable the organization to progress to more proactive phases after this. Additionally, awards available for healthcare institutions through government and sustainability networks should target foodservices to recognize achievements in this area and help build a supportive culture and motivation for this, such as those awarded by Practice Greenhealth (37). Related regulations and healthcare models that dictate the functioning of foodservices need to be realigned toward the greater goal of environmental sustainability as well as nutritious food for patients. An example of this is models whereby hospitals are able to have their own kitchens on site rather than being obliged to purchase prepared meals within a centralized production model, which was identified in several cases, and has previously been identified as a barrier to sustainable practices (12). Policy advocacy is needed to realign existing policies and standards to support sustainable foodservices; however, the communication of this value must be targeted to be most effective (38). Policy advocacy was demonstrated by hospitals in how they shared their success stories and engaged with policymakers to raise issues and advocate for more supportive high-level policy. The mechanism hospitals use for food policy advocacy is understudied in the wider literature, however, and of much value for future research to upskill hospital staff to mobilize themselves to be policy entrepreneurs and trigger systemic policy change (39). A common value within the hospital was the desire for healthy and good-quality food to support patient care, as well as providing an avenue for health-promoting messaging. This is an ideal way in which sustainable foodservices must be communicated, framing the value in terms of patient care and health, and these benefits being multi-layered, rather than purely environmental or economic. A unique finding of this research was the influence that these exemplar hospitals had on the wider community. Depending on the values of the hospital, the ability to influence wider society through sharing their work can act as an incentive to make positive changes.

This study effectively highlighted the drivers and enablers of success among hospitals with environmentally sustainable foodservices. Hospitals were recruited from a range of countries around the world, using an array of recruitment methods. Selecting exemplar cases was an appropriate method to achieve the aims as it allowed an understanding of the functioning of best cases. Participants were also in a variety of roles which allowed a diversity of perspectives to be shared. The study provides an overarching big picture view of a number of factors in play in driving environmentally sustainable foodservices in hospitals. However, we acknowledge that specific factors might play out differently in different contexts. Therefore, while the factors provide an understanding of the issues that matter, they need to be examined in any specific context and in combination with other potential factors which might not have been picked up in this study but might be appropriate in some contexts. As interviews were conducted during times of high demand on hospitals due to COVID-19 in many parts of the world, this may have limited recruitment. Additionally, the case study research, a common method of data collection is observation, although, given the travel constraints at the time and the geographical breadth of cases, this was not possible. However, reports and other documentation and in-depth interviews allowed a rich understanding of the case.

To conclude, these findings have demonstrated the institutional factors that have enabled success in achieving environmentally sustainable foodservices in hospitals internationally. While individuals have driven change, caution is needed to ensure that individuals are not solely relied upon to drive change, and instead, the healthcare system and influencing policies, regulations, and standards are crafted to support the delivery of environmentally sustainable foodservices.

## Data Availability Statement

The datasets presented in this article are not readily available because did not receive ethics or consent for sharing data. Requests to access the datasets should be directed to Stefanie Carino, stefanie.carino@monash.edu.

## Ethics Statement

The studies involving human participants were reviewed and approved by Monash University Human Research Ethics Committee. The participants provided their written informed consent to participate in this study.

## Author Contributions

SC undertook investigation and formal analysis. JP and JC verified coding and interpretation. SC wrote the original draft. All authors were involved in conceptualization and methodology and contributed to reviewing and editing.

## Funding

SC was supported by the Australian Government Research Training Program (RTP) Scholarship.

## Conflict of Interest

The authors declare that the research was conducted in the absence of any commercial or financial relationships that could be construed as a potential conflict of interest.

## Publisher's Note

All claims expressed in this article are solely those of the authors and do not necessarily represent those of their affiliated organizations, or those of the publisher, the editors and the reviewers. Any product that may be evaluated in this article, or claim that may be made by its manufacturer, is not guaranteed or endorsed by the publisher.
