# Supplementary material for: The Drivers of Environmentally Sustainable Hospital Foodservices
Source: Front Nutr. 2021 Oct 15;8:740376. doi: 10.3389/fnut.2021.740376 (PMC8554292; doi:10.3389/fnut.2021.740376)
Supplement: Supplementary file 1 [file Table_1.DOCX]

**Supplementary material**

Fig S1 – Interview protocol

| *Background*   - Firstly, could you please explain your current role and involvement with foodservices? - Tell me about the sustainable food practices at your hospital – what makes your hospital a leader in this area? |
| --- |
| *Institutional drivers*   - So now I would like to identify the key drivers for the practices, and then we will go through each one in more depth.   What do you think are the key drivers for these practices? – What has enabled these practices?   - Is there anything else you can you think of? - Prompts: - values and knowledge of the people involved? - social norms or culture of those involved? - relevant goals or incentives? - leadership that has been influential? - community expectations that has had an influence? - existing hospital policies or protocols? - local or state/national level government policies, laws or regulations involved? |
| *Understanding the institutional drivers*   - So it sounds like the key drivers here are: xxx Is that correct?   Now I’d like to understand these drivers in more depth.   - Thinking about xx,   *Background on the driver*   - Where has this come from? - What is the background to it? - How do you think this was established?   *Practicality of driver*   - How does it work in practice? - Who is involved?   *Impacts of driver*   - - What impacts have you seen as a result?   - Can you describe how things were before this driver existed?   *Repeat above questions for next driver* |
| *If the participant refers to policy as a driver*  *Policy content*   - - Tell me about the policy   Prompts:   - - What does it include?   - What doesn’t it include?   *Policy creation*   - - Tell me about the creation of this policy   Prompts:   - - Do you know who was involved?   - How was the idea sparked?   - What was the process like?   - Was there demand for this policy?   *Implementation*   - - Tell me about the implementation of the policy   Prompts:   - - How did these systems operate prior to the policy?   - How was the policy communicated?   - How were those involved trained on implementing the policy?   - How would you describe the organisation’s response to the policy?   - What has been the direct impact of the policy?   - What was needed (eg resources, structures, systems etc) to be able to effectively implement this policy?   - Have there been difficulties related to implementing this policy?   *Actors*   - - Who was involved in implementing the policy?   What has been the response/attitudes of those involved towards the policy? |
| *Barriers*   - What barriers made the hospital’s sustainable practices more difficult to achieve? - How have the drivers we have discussed had an influence on addressing those barriers? |
| *Organisational context*   - Is there anyone who has been involved in any of those practices that you may not have mentioned? - How would you say sustainable food practices at your hospital is being framed and discussed internally and externally? Is it seen as a food system issue or a healthcare sustainability issue? Or both? Or something else? - To further understand the drivers you explained, do you know of people/documents etc I could follow? Are you able to share relevant policy documents? - This interview has explored institutional drivers for sustainable foodservice. Is there anything else you would like to say on this topic before we end?   Thank you very much for your time. |
